# Long-term biochemical results after high-dose-rate intensity modulated brachytherapy with external beam radiotherapy for high risk prostate cancer

**DOI:** 10.1186/1748-717X-7-31

**Published:** 2012-03-07

**Authors:** Pedro J Prada, Lucia Mendez, José Fernández, Herminio González, Isabel Jiménez, Elisabeth Arrojo

**Affiliations:** 1 Department of Radiation Oncology, Hospital Universitario Central de Asturias, Oviedo, Spain; 2 Department of Radiation Physics, Hospital Universitario Central de Asturias, Oviedo, Spain; 3 Department of Radiation Oncology, Hospital Central de Asturias, C/Julian Claveria s/n, Oviedo 33006, (Asturias), Spain

## Abstract

**Background:**

Biochemical control from series in which radical prostatectomy is performed for patients with unfavorable prostate cancer and/or low dose external beam radiation therapy are given remains suboptimal.

The treatment regimen of HDR brachytherapy and external beam radiotherapy is a safe and very effective treatment for patients with high risk localized prostate cancer with excellent biochemical control and low toxicity.

## Introduction

Patients with clinical stage T1c, Gleason score sum 6 tumors, and prostate-specific antigen (PSA) values < 10 ng/ml have a high likelihood of disease-free survival, regardless of the treatment option chosen.

Nevertheless, we are faced with a great dilemma when we seen newly diagnosed patients with high-risk prostate cancer. Unfortunately, results of conventionally accepted therapies such as radical prostatectomy and/or standard radiotherapy have not provided these patients with good outcomes [1-3].

The need for increased dose in patients with prostate cancer was suggested by dose response observations by Pollack [4,5] and Hanks [6,7].

In an effort to improve outcomes several new radiation therapy strategies have been developed over the last decade. One approach was a combination of external beam radiotherapy (EBRT) with an intensity modulated with high dose rate (HDR) prostate brachytherapy boost.

It has been possible to increase dose, thanks to the brachytherapy advances, which allows for an increase in tumour dose (boost doses greater than 125 Gy can be safely delivered) while reducing the volume of surrounding normal tissue that is irradiated.

From a biologic perspective, the low prostate cancer α/β [8] favors a large dose per fraction in terms of cancer control. At the same time, the α/β of the rectum and bladder favors larger doses per fraction to increase the therapeutic window, thus improving control while limiting toxicity.

This study reports the long-term outcome during the PSA era for patients with high-risk prostate cancer who were treated with intensity modulated HDR boost.

## Material and methods

### Selection of patients

From June 1998 to August 2006, 252 consecutive patients were treated for high risk clinically localized prostate cancer with external beam radiation and intensity modulated HDR brachytherapy boost.

### Staging

In all cases, staging evaluation included a history and physical examination, digital rectal palpation, serum PSA, chest X-ray, bone scan, abdominal CT and/or MRI, transrectal ultrasound (TRUS), and TRUS-guided biopsy with Gleason score histologic grading. All patients were x' according to the American Joint Committee on Cancer (AJCC) 4th edition [9]. Tumour characteristics are shown in Table 1.

**Table 1:** Patient and tumor characteristic (n = 252)

Characteristics	N° Patients (%)
Stage	
≤ T2a	36 (14%)
T2b	58 (23%)
≥ T2c	158 (63%)
Geason score:	
≤ 6	109 (43%)
= 7	77 (31%)
> 7	66 (26%)
Pretreatment PSA (ng/ml)	
≤ 10	44 (17%)
10.1-20	101 (40%)
> 20	107 (43%)
Mean:20/Median 18 (2.05-59.60)	
Adjuvant hormonal ablation	
Yes	173 (69%)
No	79 (31%)
Age at diagnosis (yr)	
≤ 60	32 (13%)
61-70	131 (52%)
> 70	89 (35%)
Risk Level	
High Risk by Gleason y/o PSA	187 (74%)
High Risk by T	65 (26%)
No. Prognostic factors	
2 intermediate Risk Criteria	44 (17%)
1 High Risk Criteria	100 (40%)
2 High Risk Criteria	89 (35%)
> 2 Risk Criteria	19 (8%)
Gland Vol. Implant (cc): Mean:34/Median 31 (9-87)	

Excluded from the program were those patients who had any of the following conditions:

- Previous radiotherapy to the pelvis

- Patients with another malignant process (except skin tumour) 5 years before the diagnosis of the prostate cancer

- Recurrence of prostate tumour

- Life expectancy < 5 years

### Definition groups

Patients were considered high risk according to the Memorial Sloan Kettering group definition (clinical stage ≥ T2c or prostate-specific antigen, PSA > 20 ng/ml or Gleason score > 7 or 2-3 intermediate-risk criteria) [10].

### Hormonal therapy

In our patient population, more than half of the patients, 69% received hormonal ablative treatment (LhRh agonists + antiandrogens) for one year. It was initiated as neo-adyuvant treatment, three months before the start of radiotherapy.

### Treatment

For several years, patients diagnosed with prostate cancer have been treated at our Institution with EBRT interdigitated with two intensity modulated brachytherapy (IMBT) HDR boosts (Table 2).

**Table 2:** Treatment scheme

Weeks	External radiotherapy	Brachytherapy
1st week	2 Gy/day × 4 days	1st HDR; 5th day (11.5 Gy)
2nd week	2 Gy/day × 5 days	No
3rd week	2 Gy/day × 4 days	2nd HDR; 15th day (11.5 Gy)
4th week	2 Gy/day × 5 days	No
5th week	2 Gy/day × 5 days	No
Total dose	46 Gy/23 sessions	23 Gy/2 sessions

HDR = high dose rate (brachytherapy)

Total pelvic external beam radiation technique was 46 Gy delivered in 23 fractions of 2 Gy over 4.5 weeks. All patients were treated using 18-MV photons. No external radiation was delivered the same day of the HDR brachytherapy procedure (day 5 and 15). Total treatment time including the HDR boost was over a period of 5 weeks. All fields were treated daily. Isocentric technique was used and all fields were equally weighted. The portals used covered the prostate, seminal vesicles, and the periprostatic tissues with a margin of at least 1 cm. The tumour volume was encompassed in the fields by the 100% isodose line ± 5%.

Brachytherapy procedures were done under spinal anesthesia. The dose administered in each application was 11.5 Gy, except in the first group of patients treated (17%) in whom the dose applied varied between 10.5 and 11. The target volume of the implant was the prostate gland + 5 mm peri-prostatic area and medial aspects of seminal vesicle. All patients were discharged from the center the same day of the procedure between 6-8 hours of implantation.

The total combined BED dose ranged from 292 Gy to 366 Gy based upon a α/β ratio of 1.2 [8].

### Toxicity

Patients were followed with symptom assessment and PSA determinations every 3 months for the first year, every 6 months for the second year and yearly thereafter.

Toxicity was reported according to the Common Toxicity Criteria for Adverse Event, Version 4.0 (CTAE v4.02) by the National Cancer Institute. Chronic toxicity was defined as those symptoms which persisted or presented beyond 6 months. Potency was defined as the ability to achieve an erection that was sufficient for intercourse.

Toxicity and sexual side-effects was scored by the physician.

### Statistical considerations

Distant metastatic disease was defined by an imaging study or physical examination that demonstrated cancer outside of the prostate and its regional nodes. Failure in cause-specific survival (CSS) analyses was defined as death due to prostate cancer. Failure in tumour-free survival (TFS) analyses was represented as detection of local and/or systemic tumour relapse, and bNED for no clinical and biochemical evidence of disease. Overall survival reflected all deaths, cancer-related or otherwise. To assess the local relapse, sextant prostate biopsies were taken in patients with no metastatic disease if they did not refuse this procedure. Biochemical failure was defined according to the "Phoenix definition" [11] consensus panel statement. Estimated likelihood of events was calculated by the Kaplan-Meier method from the time of completion of radiotherapy. The statistical significance of the difference between estimated event-free

Curves were calculated with the long-rank test. Multivariate analysis was performed using the Cox proportional hazards model [12]. Statistical analyses were performed with SPSS version 17.0 (SPSS Inc, Chicago, IL).

## Results

### Clinical characteristics

All patients treated in the protocol were included for analysis and completed the planned course of radiation. All patients have been seen in follow up.

The high risk group, as defined in this study, represented 100% of the patient population (74% were high risk by Gleason and/or PSA and 26% by T-stage) (Table 1).

The median V100, V90, V150 and V200 (% volume of CTV receiving 100% of prescription dose) were respectively 93.73% (96.82-85.81), 97.90% (99.80-91.79%), 21.02% (38.17-12.64) and 5.97% (9.41-3.30). The median D90 (The dose that covers 90% volume of CTV) was 12.04 Gy (18.38-9.45). Maximum urethral point dose was 12.66 Gy (16.36 Gy-9.25 Gy) and maximum rectal point dose was 9.56 Gy (14.09 Gy-8.2 Gy).

### Oncologic endpoints

Of all 252 patients, 51 had evidence of biochemical relapse, 42 had clinical relapse and 12 died from prostate cancer; 35 patients died of other illnesses. Mean and median follow-up for all patients were 77 and 74 months respectively with a range of 12 to 142 months.

In the 51 patients with a biochemical failure, the median time to PSA failure was 38 (5-80) months, with 55% failing within 3 years and 98% within 6 years. In patients with no biochemical failure, the mean and median PSA level after treatment was 0.10 and 0.03 (0.0-1.2) ng/ml, the last follow-up PSA levels were ≤ 0.2 ng/ml in 90%, < 1 ng/ml in 99.6% and 1.2 ng/ml in a patient.

**Figure 1:**
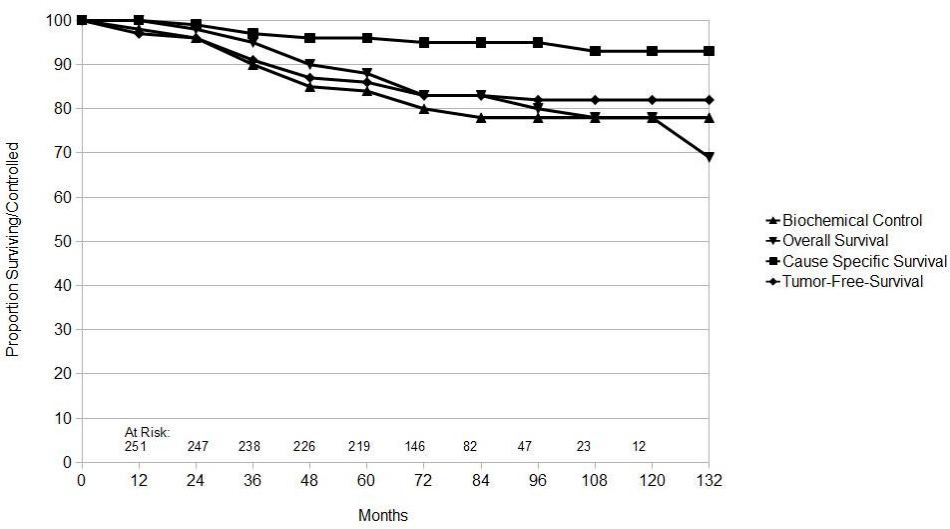
**Actuarial analysis of all 252 patients for Cause specific survival, tumor-free survival, biochemical control and overall survival**.

The 5 and 10 years for biochemical control were 84% and 78% (SD ± 2%), whereas in tumor-free survival (TFS) they were 86% and 82% (SD ± 2%) at 5 and 10 years respectively. The 10 year cause specific survival was 93% (SD ± 2%) with 99% (SD ± 1%) of patients being free of local recurrence. The overall survival according to Kaplan-Meier estimates was 88% and 78% (SD ± 4%) at 5 and 10 years respectively (Figure 1).

Characteristics used for multiple regression analyses to correlate with biochemical failure were: clinical T-classification, Gleason score, pretreatment PSA, age, brachytherapy dose level, prostate volume and hormonal ablative treatment.

The multivariate Cox regression analyses identified, Gleason score as independent prognostic factors for biochemical failure.

**Figure 2:**
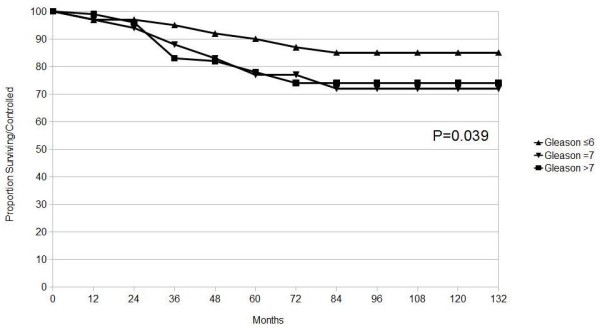
**Actuarial analysis of biochemical control by Gleason score**. P value generated from Log-Rank.

The 10-year actuarial biochemical control stratified by Gleason score was 85%, 72% and 74% for patients with Gleason score of ≤ 6, 7 and > 7, respectively (P = 0.039) (Figure 2).

**Figure 3:**
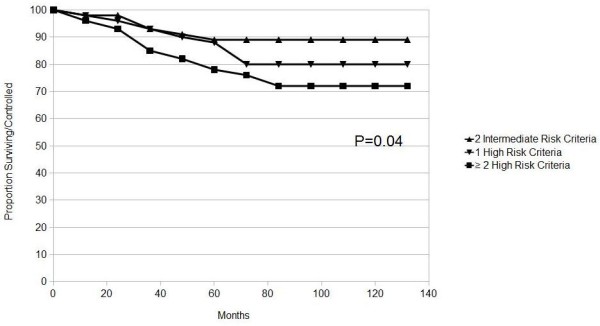
**Actuarial analysis of biochemical control by poor prognostic factors groups**. P value generated from Log-Rank.

The 10-year actuarial biochemical control was 89% for patients with two intermediate risk criteria, 80% with one high risk criteria and 72% for patients with 2-3 high risk criteria (P = 0. 04) (Figure 3).

**Figure 4:**
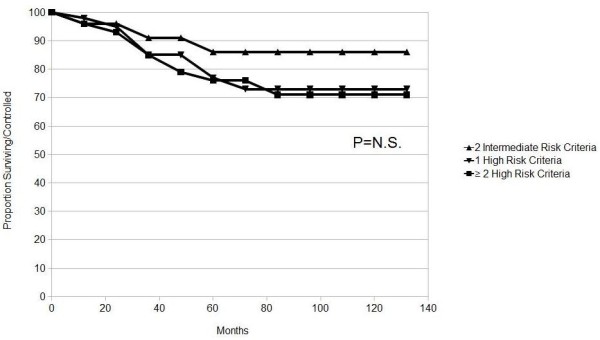
**Actuarial analysis of biochemical control by poor prognostic factors groups in patients classified as high risk only by Gleason and/or PSA, no by T-stage**. P value generated from Log-Rank.

The 10-year actuarial biochemical control in patients classified as high risk only by Gleason and/or PSA, no by T-stage, was 86% for patients with two intermediate risk criteria, 73% with one high risk criteria and 71% for patients with 2 high risk criteria (P = 0. 45) (Figure 4).

Mean age was 67 years (range 49-78). The actuarial analysis of biochemical control at ages less than 60, 60 to 69 and greater than 69 years demonstrated no significant difference. As younger and older patients benefited equally (P = 0.148).

The actuarial biochemical control was 80% and 74% (P = 0.173), in patients who received hormones and in those who did not.

All other variables (pretreatment PSA P = 0.898, T-classification (p = 0.273), brachytherapy dose level P = 0.492, and volume P = 0.615) were statistical no significant for biochemical failure.

Metastatic disease developed in 42 patients and the distant metastatic rate at 10-years was 14%. Multivariate analysis showed that Gleason score was the only factor to significantly affect distant metastases, with 10-year rates of 7%, 21% and 17% for scores of ≤ 6, 7 and > 7, respectively (P = 0.014).

The 10-year actuarial tumour-free survival (TFS) was 90%, 73% and 78% for patients with Gleason scores of ≤ 6, 7 and > 7, respectively (P = 0.020).

### Acute and chronic urinary toxicity

Moderate increase in urinary frequency and tract pain (urethritis) occurred at the end of the treatment. At 6 month genitourinary grade I toxicity was 6% (increase in urinary frequency and dysuria. Moderate symptoms). Acute grade 1 urinary retention requiring a temporary post-implant bladder catheter was seen in 4 (1.6%) patients.

Grade 4 or 5 late toxicity was not detected in any patients. Thirteen patients (5%) showed, increase in urinary frequency and dysuria at 12 month.

A urethral stricture after treatment was observed in seven patients (2.7%). Four patients required intermittent bladder catheterization and endoscopic urethrotomy was required in three patients.

No patients reported incontinence after treatment.

### Gastrointestinal toxicity

Acute gastrointestinal toxicity grade II was 3%, consisting in increase of 4-6 stools per day over baseline and mucus in stool, no patients developed grade 3 toxicity. At six months gastrointestinal grade II toxicity was 1.6%.

At 12 months 99% of patients reported no change in bowel function. Intermittent rectal bleeding without systemic repercussions was reported in 5 patients (2%). No patients with perineal pain were reported.

### Sexual function

Of the 131 (52%) patients who were potent preoperatively and hormonal manipulation was not used, 72% were potent postoperatively but only 68% the patients were potent when hormonal manipulation was used. Potency was defined as the ability to achieve an erection that was sufficient for intercourse.

### Correlation between post implant dosimetry and toxicity

Characteristics used for multiple logistic-regression analyses which correlates probability of acute and chronic urinary toxicity were: pre-treatment prostate volume, year of implant, number of needles implanted, brachytherapy dose, hormonal ablative treatment, maximum urethral dose.

The multivariate logistic-regression analyses showed than patients with high urethral dose after established brachytherapy technique are more likely to suffer acute urethritis (P = 0.018).

## Discussion

The importance of dose escalation, has been well documented, high radiation doses improve biochemical and clinical results for prostate cancer patients [4-7]. Kuban et al. reported an improvement in biochemical control of 78% for doses of 78 Gy, vs 59% for the 70 Gy arm [13].

The combination of EBRT and HDR brachytherapy allows the delivery of very high biologic equivalent doses to the prostate not achievable by intensity modulated treatments (IMRT) with image guided adaptive radiotherapy (IGART) techniques.

Based on these principles, since June 1998 we have been performing High dose rate brachytherapy boost for prostate cancer.

The high risk group in our report represented 100% of the patient population and the results of this combined therapy (EBRT + HDR boost) at 10-years are promising, with a biochemical control rate of 78%, cause specific survival of 93%, overall survival of 78%, and freedom from distant metastases of 86%. The 10-year actuarial biochemical control in patients classified as high risk only by Gleason and/or PSA, no by T-stage, was 86% for patients with two intermediate risk criteria, 73% with one high risk criteria and 71% for patients with 2 high risk criteria.

The results presented here for high risk group of patients, are superior to the series on radical prostatectomy and standard radiotherapy therapies published in the literature.

The Memorial Sloan Kettering reported in unfavorable risk cases 5-year PSA relapse-free survival rate for 81 Gy the 67% versus 43% for 75.6 Gy and 21% for 64.8 to 70.2 Gy [14].

Hanks et al. observed that patients with unfavorable disease (Gleason ≥ 8 and PSA ≥ 20 ng/ml) treated with three-dimensional conformal radiation therapy (3D CRT) a dose of 76 Gy achieved only 26% in the 5-year PSA relapse-free survival rate [15]. Dearnaley et al., Sathya et al. and Zietman et al. reported similar results [16-18].

Our study shows an advantage to high-dose over conventional-dose conformal radiation in terms of freedom from biochemical failure for men with high risk prostate cancer.

Similar observations were reported by other institutions using conformal high dose rate brachytherapy. Martinez et al. [19] reported 5-year actuarial biochemical control rate of 85% for patients with 1 poor prognostic factor, 75% for 2 and 50% for all 3. Galalae et al. reported 8-year bNED survival rate (free of clinical or biochemical evidence of disease) in the high-risk prognostic group of 64% [20]. Dattoli et al. [21] and Mate et al. [22] reported similar results. A recent report Stock et al. reported 8-year actuarial biochemical control rate of 73% for patients with Gleason score 8-10 prostate cancer [23].

On the other hand, surgery is not the best treatment for high risk patients. Catalona et al. [24] Studied 3478 men with tumors of clinical stages T1-T3 N0 M0 followed for an average of 65 months after radical retropubic prostatectomy. Actuarial 10-year biochemical progression-free probabilities were 59% for cT2b-c, 15% for cT3 disease and 50% for Gleason sum 4 + 3 and 32% for Gleason 8-10 disease. Actuarial 10-year biochemical progression-free probabilities were 49% for PSA greater than 10 ng/ml.

The Johns Hopkins group reported similar results [25,26]. Kermen and Miles [27] reported a 5-year bNED rate of 54% after radical prostatectomy. Surgery results of Multi-institutional pooled analysis in men with locally advanced prostate cancer [2] published a 2.2-year of biochemical control rate of 16%.

The Memorial Sloan Kettering Center group analyzed the oncologic outcome after laparoscopy radical prostatectomy, 8-year probably of freedom from progression for high risk cancer was 53% [28].

In patients with high risk prostate cancer, the 10-year biochemical control was 16-54% for prostatectomy treated patients compared to 86-71% for our combined conformal EBRT with HDR boost.

Despite the wide diffusion of laparoscopic radical prostatectomy and robot-assisted laparoscopic radical prostatectomy, only few studies comparing the results of these techniques with the retropubic radical prostatectomy. The systematic review of the literature performed by Ficarra et al. [29], were not sufficient to prove the superiority of any surgical approach in terms of functional and oncologic outcomes.

Gleason score was in our paper the most significant predictor of biochemical failure and developing distant metastases. Other groups reported similar results [19,21,23].

Hormonal ablative treatment did not improved the outcome in the present analysis, this corroborate the findings of previous studies [21,30,31]. All prospective, randomized trials show a positive outcome of adding hormonal therapy [32-34] but these studies were done with low radiation doses (68-70 Gy), the BED equivalent of 70 Gy was 129 Gy, much lower than BED in our series (BED dose ranged from 292 Gy to 366 Gy).

The low toxicity observed in our series, despite the high radiation doses delivered, was the result of carefully executed real-time brachytherapy technique [35]. Urinary and Gastrointestinal complications rates were in concordance with the experience of other institutions using conformal high doses rate brachytherapy [19-22] and favourably with other 3-D conformal radiotherapy escalating series [14,15].

The cause specific survival at 10-year of 93% and biochemical control of 78% demonstrate the effectiveness of the described radiotherapy regime and the high curative potential of this therapy protocol.

## Conclusions

The present studies shows that when men with high risk clinically localized prostate cancer are treated with EBRT interdigitated with two HDR Ir-192 brachytherapy boost, allows us to administer the highest possible dose to the prostate and the lowest dose to the surrounding healthy structures, achieved excellent results in terms of local and biochemical control, decrease the toxicity and the overall treatment time by at least 3 weeks compared to 3-D conformal radiation therapy and intensity modulated.

In summary this treatment regimen is a safe and very effective for patients with high risk localized prostate cancer and represents a considerable improvement over standard surgical and radiotherapy modalities.

## Abbreviations

AJCC: American Joint Committee on Cancer; BED: Biologically effective dose; BTm: Brachytherapy; bNED: No clinical and biochemical evidence of disease; CTV: Clinical target volume; CSS: Cancer specific survival; CT: Computed tomography; D90: The dose that covers 90% volume of CTV; 3D CRT: Three-dimensional conformal radiation therapy; EBRT: External beam radiotherapy; GU: Genitourinary; HDR: High dose rate; IMRT: Intensity modulated radiotherapy; IGART: Image guided adaptive radiotherapy; PSA: Serum prostate-specific antigen; PTV: Planning target volume; CTAE v4.02: Common Toxicity Criteria for Adverse Event, Version 4.0 by the National Cancer Institute; SPSS: Statistical analysis SPSS; SD: Standard desviations; TFS: Tumour-free survival; TRUS: The trans-rectal ultrasound; V100, V90, V150 and V200: (% volume of CTV receiving 100% of prescription dose).

## Competing interests

The authors declare that they have no competing interests.

## Authors' contributions

PJP: conception and design. LM acquisition of data. JF analysis of data. HG revising references. IJ alignment and drafted the manuscript. EA acquisition of data. All authors read and approved the final manuscript.
